# The Who, Why, and How of Small-Molecule Production in Invertebrate Microbiomes: Basic Insights Fueling Drug Discovery

**DOI:** 10.1128/mSystems.00186-17

**Published:** 2018-03-13

**Authors:** Jason C. Kwan

**Affiliations:** a Division of Pharmaceutical Sciences, School of Pharmacy, University of Wisconsin—Madison, Madison, Wisconsin, USA

**Keywords:** genome reduction, metagenomics, metatranscriptomics, natural products/secondary metabolites, symbiosis, synthetic biology

## Abstract

Bacteria have supplied us with many bioactive molecules for use in medicine and agriculture. However, rates of discovery have decreased as the biosynthetic capacity of the culturable biosphere has been continuously mined for many decades. The as-yet-uncultured biosphere is likely to hold far greater biosynthetic potential, especially where ecological niches favor the selection of therapeutically useful bioactivities. I outline here how metagenomics and other systems biology approaches can be used to gain insight into small-molecule biosynthesis and the selective forces which shape it. I also argue that we need a greater understanding of the function of small molecules in complex microbiomes and rational synthetic biology methods to functionally reconstruct large biosynthetic pathways in heterologous hosts.

## Abstract (precis)

Bacteria have supplied us with many bioactive molecules for use in medicine and agriculture. However, rates of discovery have decreased as the biosynthetic capacity of the culturable biosphere has been continuously mined for many decades.

## PERSPECTIVE

Nature is an accomplished synthetic chemist, and a large fraction of bioactive molecules used today in medicine and agriculture are either evolved small molecules or were inspired by such agents (1). Natural selection favors the generation of compounds that improve the odds of survival, and these compounds can also be therapeutically useful for humankind if their mechanism of action impacts disease mechanisms. For example, many bacteria produce molecules that inhibit the growth of rival species or fungi, and we use many of these as antibacterial or antifungal treatments. Likewise, some plants produce toxic compounds that protect them from grazing animals, and many such compounds (for example, paclitaxel [originally named taxol]) are now used as cancer therapeutics. However, evolution also works against us—the widespread use of antibiotics in human medicine and agriculture selects for the propagation of resistance genes (2), some of which evolved long before antibiotics were used by humans (3), to confer self-resistance on antibiotic-producing organisms. In the case of antibiotics, recent decades have seen a precipitous drop in discovery rates (4), as soil-derived culturable microorganisms and synthetic chemistry programs have not yielded the number of drug leads originally envisioned. If we are to discover more drugs from nature, it would be wise to explore novel environments and parts of the tree of life that have been undersampled and to gain a greater understanding of the evolutionary and ecological forces that favor bioactive small-molecule production.

My research group and others have been exploring the biosynthetic potential of the as-yet-uncultured biosphere, using culture-independent sequencing techniques such as metagenomics and metatranscriptomics. Metagenomics and other systems biology methods have started to illuminate the true scope of microbial biodiversity on Earth (5, 6). The biosynthetic pathways that produce small molecules are widely distributed in bacteria (7), and they are thought to mediate complex interactions in nature, known as the “parvome” (8, 9). Although parts of the parvome—for example, quorum-sensing systems—have been studied, we currently lack a systematic understanding of chemical interactions in complex microbial communities. This stems from an inability to describe microbiome behavior at the level of individual species or strains—in other words, who is doing what, and why? Metatranscriptomics can be used fairly easily to determine gene expression trends in aggregate, but without knowing which species each transcript belongs to, changes in species abundance cannot be distinguished from expression changes. Increasingly, it is understood that genomes vary among environmental bacteria, and the complete set of genetic capabilities exhibited by all strains in a species can be considered the “pan-genome” (10). Accordingly, we have begun to examine transcriptome sequencing (RNA-seq) and metagenomics data from the same environmental sample, to allow the *de novo* assembly of novel genomes and to avoid problems with strain variability when aligning RNA-seq reads to DNA contigs (11, 12).

**FIG 1:**
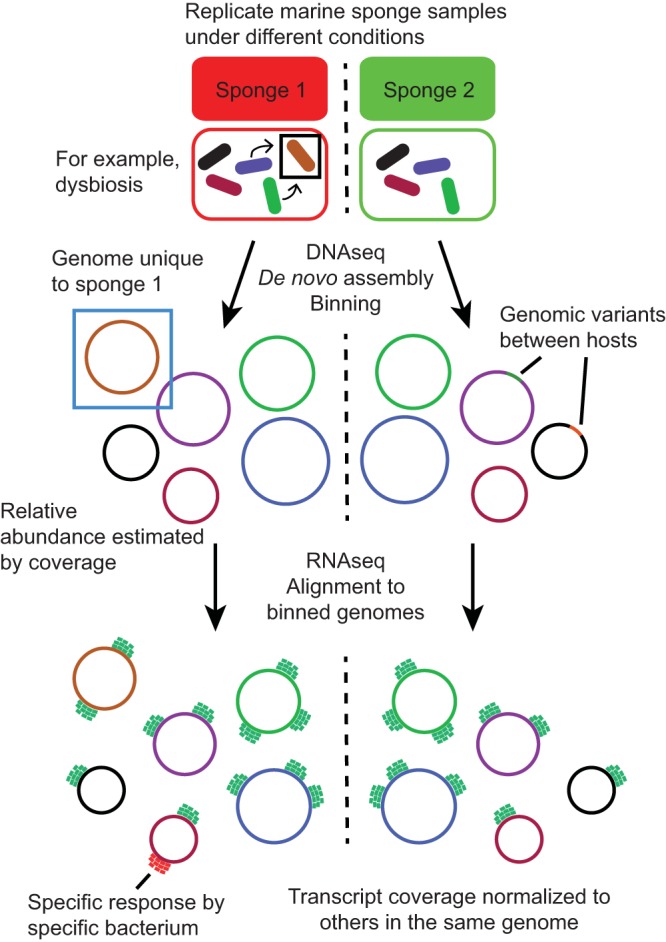
Investigating microbiome function with shotgun metagenomics and metatranscriptomics. The effects of different environmental conditions, for example, dysbioses in marine sponges, can be studied through a combination of metagenomics and metatranscriptomics. Individual bacterial genomes can be extracted through metagenomic assembly, followed by binning (who is there and what they can potentially do). Integrating metatranscriptomics with binned genomes allows transcripts to be normalized on a per-genome basis, reducing the effects of genome copy number changes on relative expression quantification. This allows us to determine who is responding to the environmental change and how. DNAseq, DNA sequencing; RNAseq, RNA sequencing.

In such matched DNA and RNA data sets, the accurate assignment of metagenomic contigs to species-level “bins” allows transcript expression to be quantified relative to housekeeping genes in the same genome, normalizing for changes in genome copy number between samples (Fig. 1). We are currently using these techniques to study the behavior of the marine sponge microbiomes in response to dysbiosis. Sponges can have highly complex microbiomes containing hundreds of microbial species that often include highly divergent, novel species, making binning challenging. Semimanual methods of binning are too labor-intensive in these systems, and many of the automatic methods fail because they do not separate the host sponge genome. Other methods rely on coassembly of many samples, but the quality of coassemblies is degraded by interstrain variability between samples. Vertically transmitted symbionts are expected to exhibit sequence drift in different hosts (see below), and so coassembly of pooled samples can result in highly fragmented and chimeric contigs. We therefore have developed our own binning pipeline (26) so that highly complex host-associated metagenomes can be automatically and reproducibly analyzed. With accurate binning, combined DNA and RNA sequencing can be used to follow expression patterns of each microbe in a microbiome, and behaviors can be compared under different conditions. Such studies may well shed light on the environmental stimuli that initiate small-molecule synthesis in the environment. We will, however, probably require new analysis and modeling techniques to truly understand the higher-order interactions and emergent behavior of whole microbiomes.

In the absence of a systematic understanding of microbiome function, my own research group has focused on systems where there is a clear ecological rationale for chemical defense. In particular, we have investigated several marine invertebrates that are sessile and/or lack physical defenses against predation and are known to harbor cytotoxic molecules, often made by a microbial symbiont rather than the host. The existence of such symbiotic relationships based on small-molecule production implies that the small molecule has served a useful ecological function over evolutionary timescales. For example, we found evidence that the biosynthetic pathway for the patellazoles, picomolar cytotoxins isolated from the tunicate *Lissoclinum patella*, has been present in the genome of the producing symbiont for at least 6 million years (13, 14).

It is our view that the most important bioactive compounds will be found in such ecological niches where they have been honed by strong selective pressures for prolonged periods of time. However, the symbiotic environment also conspires to make bacterial symbionts difficult to culture. While selection pressure to maintain biosynthetic capability for protective or defensive small molecules is strong in symbionts (13, 15), pressure to maintain basic metabolic functions needed for independent growth is weakened because of the hospitable and stable host environment (16). Over evolutionary timescales, this altered selection profile and a population structure where small numbers of symbiont cells are isolated in one host individual lead to the progressive degradation of gene sequences until they become nonfunctional pseudogenes and are eventually deleted (16). After a prolonged period of time, this “genome reduction” process yields very tiny genomes (~<500 kbp) that cannot support life outside the host. We have therefore used shotgun metagenomics extensively to gain insight into the life of symbiotic bacteria that make small molecules.

**FIG 2:**
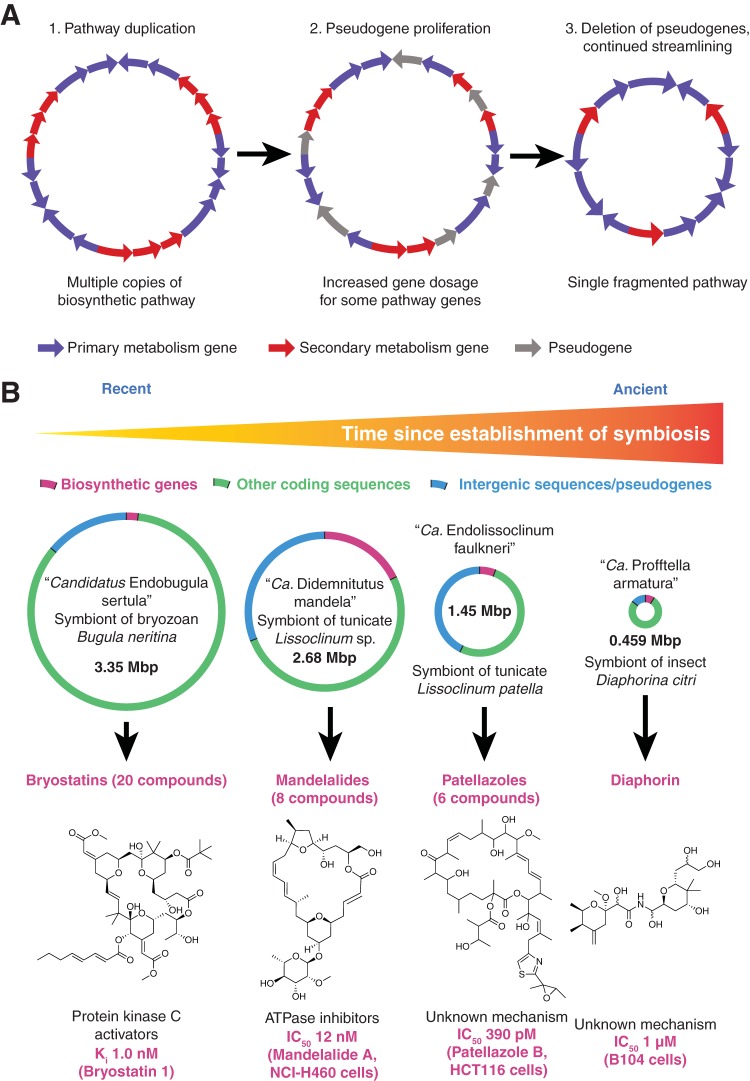
(A) Model for the transition from biosynthetic pathway duplication shortly after establishment of symbiosis to pathway fragmentation frequently observed in older symbionts (18). Early in the symbiosis, selection pressure for increased compound production could lead to pathway duplication. However, loss of DNA repair pathways and facile fixation of mutations due to frequent population bottlenecks give rise to sequence drift and proliferation of pseudogenes. All but lethal mutations accumulate, lowering the gene dosage of each repeated gene in the pathway. Eventually, one copy of each pathway gene will remain because further loss would impact the survival of the host. The remaining copies will not necessarily originate from the same repeat, leaving a single fragmented pathway. (B) Bioactive, and presumably defensive, compounds are produced by symbionts on a continuous spectrum of genome reduction, including the bryostatins (19), mandelalides (15), patellazoles (13), and diaphorin (25). In the early stages of genome reduction, coding density decreases, and at least in the case of “*Ca*. Didemnitutus mandela,” biosynthetic gene cluster copy number increases. Intergenic sequences are progressively deleted, as more and more functional genes are also degraded and deleted, until symbionts possess dense, tiny genomes. IC_50_, 50% inhibitory concentration.

We recently used metagenomics to describe the genome of a bacterial symbiont in the phylum *Verrucomicrobia* that exemplifies this dichotomy between strong selection for secondary metabolites and weak selection for more basic functions (15). “*Candidatus* Didemnitutus mandela” lives within a marine tunicate and produces cytotoxic compounds called mandelalides (17). Its genome contains relatively few full-length genes with recognizable functions, and most of the genome is littered with either short hypothetical genes of unknown purpose or truncated forms of homologs in the closest known relative (“pseudogenes”). Despite these clear signs of genome reduction, the *mnd* pathway for the production of mandelalides is repeated seven times in the chromosome, collectively accounting for almost 20% of its total length. This likely indicates pressure for greater production through increased gene dosage. After symbionts are restricted to living within their host, they become genetically isolated and subject to extreme population bottlenecks when only a few bacterial cells are passed vertically to the host’s offspring. In this setting, mutations accumulate because they cannot be corrected by horizontal transfer among a large population, eventually leading to the loss of genes not immediately required for the symbiosis, including DNA repair pathways. “*Ca*. Didemnitutus mandela” has lost the ability to carry out homologous recombination, and consequently, a number of single nucleotide polymorphisms (SNPs) and deletions in some of the *mnd* repeats have become fixed through population bottlenecks and cannot be corrected. This process of degradation is likely to continue until only one copy of each *mnd* gene remains. Complete loss of the pathway is unlikely because hosts devoid of symbiont protection would lose their selective advantage. Many symbionts have been found to possess biosynthetic pathways that are fragmented throughout the genome, in contrast to the contiguous gene “clusters” found in free-living bacteria (18). This fragmentation could have arisen after early duplication events, as in “*Ca*. Didemnitutus mandela,” followed by progressive degradation of pathway genes until each occurred as single copies originating from repeats in different locations (Fig. 2A).

Importantly, it is not always obvious why particular bacterial symbionts are intractable to laboratory culture. For example, we recently sequenced the genome of “*Candidatus* Endobugula sertula,” a symbiont of the bryozoan *Bugula neritina* that produces defensive compounds called bryostatins (19). Bryostatins are potent protein kinase C activators that have been evaluated in many clinical trials for cancer and HIV infection, but the isolation of 18 g of bryostatin 1 requires the collection of 10,000 gal of *Bugula neritina* (20). Despite many attempts, “*Ca*. Endobugula sertula” has never been cultured. However, the genome of this symbiont does not show signs of ongoing genome reduction, and “*Ca*. Endobugula sertula” appears to be a recent symbiont that retains capability for horizontal transmission between hosts (Fig. 2B) (11). Many other promising compounds are made by uncultured microbes, such as anticancer drug ET-743 (21), and all suffer from similar “supply problems” unless a cultured source can be identified or a synthetic route devised. In the case of bryostatins, a scalable synthesis has only recently been developed 35 years after the compounds were discovered (22). Bryostatins could be recollected or synthesized in amounts justified by initial biological findings, but rarer, and potentially even more clinically significant, agents are unlikely to be developed to this extent.

Heterologous expression of pathways might offer an alternate means of supplying novel compounds from unculturable sources, but this work is far from trivial. Thus far, such efforts have been limited to hosts that are presumably related to the producer (23) or to relatively short pathways (24). Expression of highly complex pathways, such as polyketide synthase (PKS) systems with lengths up to ~100 kbp and proteins up to ~1 MDa, will require extensive refactoring and codon optimization. As the price of gene synthesis continues to decrease, synthetic biology methods could be employed to reconstitute such pathways. To be broadly useful, synthetic biology efforts should focus on determining design rules to ensure efficient transcription, translation, and folding of large protein components in arbitrary hosts. This is currently challenging because the causes of failure in heterologous expression experiments are not well defined, and we lack methods to diagnose problems; this is especially true for large modular proteins with multiple enzymatic domains (such as in PKS pathways). In my view, these fundamental knowledge gaps represent a major roadblock in using metagenomics for drug discovery and development programs. In the coming years, my research group will be focused on solving this roadblock, using synthetic biology to establish a rational “design-build-test” loop to both identify problems in transcription, translation, and folding and determine rules for the *de novo* design of functional versions of PKS genes. My ultimate aim is to allow the seamless use of metagenomic sequencing information for the functional expression of complex pathways in heterologous hosts, thus removing the limit of “unculturability” from drug discovery in the near future.

## References

[1] Newman DJ, Cragg GM 2016 Natural products as sources of new drugs from 1981 to 2014. J Nat Prod 79:629–661. doi:10.1021/acs.jnatprod.5b01055. 26852623

[2] Witte W 1998 Medical consequences of antibiotic use in agriculture. Science 279:996–997. doi:10.1126/science.279.5353.996. 9490487

[3] D’Costa VM, King CE, Kalan L, Morar M, Sung WWL, Schwarz C, Froese D, Zazula G, Calmels F, Debruyne R, Golding GB, Poinar HN, Wright GD 2011 Antibiotic resistance is ancient. Nature 477:457–461. doi:10.1038/nature10388. 21881561

[4] Boucher HW, Talbot GH, Bradley JS, Edwards JE, Gilbert D, Rice LB, Scheld M, Spellberg B, Bartlett J 2009 Bad bugs, no drugs: no ESKAPE! An update from the Infectious Diseases Society of America. Clin Infect Dis 48:1–12. doi:10.1086/595011. 19035777

[5] Thompson LR, Sanders JG, McDonald D, Amir A, Ladau J, Locey KJ, Prill RJ, Tripathi A, Gibbons SM, Ackermann G, Navas-Molina JA, Janssen S, Kopylova E, Vázquez-Baeza Y, González A, Morton JT, Mirarab S, Zech Xu Z, Jiang L, Haroon MF, Kanbar J, Zhu Q, Jin Song S, Kosciolek T, Bokulich NA, Lefler J, Brislawn CJ, Humphrey G, Owens SM, Hampton-Marcell J, Berg-Lyons D, McKenzie V, Fierer N, Fuhrman JA, Clauset A, Stevens RL, Shade A, Pollard KS, Goodwin KD, Jansson JK, Gilbert JA, Knight R, Earth Microbiome Project Consortium 2017 A communal catalogue reveals Earth’s multiscale microbial diversity. Nature 551:457–463. doi:10.1038/nature24621. 29088705 PMC6192678

[6] Parks DH, Rinke C, Chuvochina M, Chaumeil PA, Woodcroft BJ, Evans PN, Hugenholtz P, Tyson GW 2017 Recovery of nearly 8,000 metagenome-assembled genomes substantially expands the tree of life. Nat Microbiol 2:1533–1542. doi:10.1038/s41564-017-0012-7. 28894102

[7] Cimermancic P, Medema MH, Claesen J, Kurita K, Wieland Brown LC, Mavrommatis K, Pati A, Godfrey PA, Koehrsen M, Clardy J, Birren BW, Takano E, Sali A, Linington RG, Fischbach MA 2014 Insights into secondary metabolism from a global analysis of prokaryotic biosynthetic gene clusters. Cell 158:412–421. doi:10.1016/j.cell.2014.06.034. 25036635 PMC4123684

[8] Davies J, Ryan KS 2012 Introducing the parvome: bioactive compounds in the microbial world. ACS Chem Biol 7:252–259. doi:10.1021/cb200337h. 22074935

[9] Kelsic ED, Zhao J, Vetsigian K, Kishony R 2015 Counteraction of antibiotic production and degradation stabilizes microbial communities. Nature 521:516–519. doi:10.1038/nature14485. 25992546 PMC4551410

[10] Medini D, Donati C, Tettelin H, Masignani V, Rappuoli R 2005 The microbial pan-genome. Curr Opin Genet Dev 15:589–594. doi:10.1016/j.gde.2005.09.006. 16185861

[11] Miller IJ, Vanee N, Fong SS, Lim-Fong GE, Kwan JC 2016 Lack of overt genome reduction in the bryostatin-producing bryozoan symbiont, “*Candidatus* Endobugula sertula.” Appl Environ Microbiol 82:6573–6583. doi:10.1128/AEM.01800-16. 27590822 PMC5086551

[12] Miller IJ, Weyna TR, Fong SS, Lim-Fong GE, Kwan JC 2016 Single sample resolution of rare microbial dark matter in a marine invertebrate metagenome. Sci Rep 6:34362. doi:10.1038/srep34362. 27681823 PMC5041132

[13] Kwan JC, Donia MS, Han AW, Hirose E, Haygood MG, Schmidt EW 2012 Genome streamlining and chemical defense in a coral reef symbiosis. Proc Natl Acad Sci U S A 109:20655–20660. doi:10.1073/pnas.1213820109. 23185008 PMC3528492

[14] Kwan JC, Schmidt EW 2013 Bacterial endosymbiosis in a chordate host: long-term co-evolution and conservation of secondary metabolism. PLoS One 8:e80822. doi:10.1371/journal.pone.0080822. 24324632 PMC3851785

[15] Lopera J, Miller IJ, McPhail KL, Kwan JC 2017 Increased biosynthetic gene dosage in a genome-reduced defensive bacterial symbiont. mSystems 2:e00096-17. doi:10.1128/mSystems.00096-17. 29181447 PMC5698493

[16] McCutcheon JP, Moran NA 2011 Extreme genome reduction in symbiotic bacteria. Nat Rev Microbiol 10:13–26. doi:10.1038/nrmicro2670. 22064560

[17] Nazari M, Serrill JD, Wan X, Nguyen MH, Anklin C, Gallegos DA, Smith AB III, Ishmael JE, McPhail KL 2017 New mandelalides expand a macrolide series of mitochondrial inhibitors. J Med Chem 60:7850–7862. doi:10.1021/acs.jmedchem.7b00990. 28841379 PMC5702619

[18] Miller IJ, Chevrette MG, Kwan JC 2017 Interpreting microbial biosynthesis in the genomic age: biological and practical considerations. Mar Drugs 15:165. doi:10.3390/md15060165. PMC5484115 28587290

[19] Trindade-Silva AE, Lim-Fong GE, Sharp KH, Haygood MG 2010 Bryostatins: biological context and biotechnological prospects. Curr Opin Biotechnol 21:834–842. doi:10.1016/j.copbio.2010.09.018. 20971628 PMC4497553

[20] Schaufelberger DE, Koleck MP, Beutler JA, Vatakis AM, Alvarado AB, Andrews P, Marzo LV, Muschik GM, Roach J, Ross JT 1991 The large-scale isolation of bryostatin 1 from *Bugula neritina* following current good manufacturing practices. J Nat Prod 54:1265–1270. doi:10.1021/np50077a004. 1800630

[21] Schofield MM, Jain S, Porat D, Dick GJ, Sherman DH 2015 Identification and analysis of the bacterial endosymbiont specialized for production of the chemotherapeutic natural product ET-743. Environ Microbiol 17:3964–3975. doi:10.1111/1462-2920.12908. 26013440 PMC4618771

[22] Wender PA, Hardman CT, Ho S, Jeffreys MS, Maclaren JK, Quiroz RV, Ryckbosch SM, Shimizu AJ, Sloane JL, Stevens MC 2017 Scalable synthesis of bryostatin 1 and analogs, adjuvant leads against latent HIV. Science 358:218–223. doi:10.1126/science.aan7969. 29026042 PMC5714505

[23] Iqbal HA, Low-Beinart L, Obiajulu JU, Brady SF 2016 Natural product discovery through improved functional metagenomics in *Streptomyces*. J Am Chem Soc 138:9341–9344. doi:10.1021/jacs.6b02921. 27447056 PMC5469685

[24] Schmidt EW, Nelson JT, Rasko DA, Sudek S, Eisen JA, Haygood MG, Ravel J 2005 Patellamide A and C biosynthesis by a microcin-like pathway in Prochloron didemni, the cyanobacterial symbiont of Lissoclinum patella. Proc Natl Acad Sci U S A 102:7315–7320. doi:10.1073/pnas.0501424102. 15883371 PMC1091749

[25] Nakabachi A, Ueoka R, Oshima K, Teta R, Mangoni A, Gurgui M, Oldham NJ, van Echten-Deckert G, Okamura K, Yamamoto K, Inoue H, Ohkuma M, Hongoh Y, Miyagishima SY, Hattori M, Piel J, Fukatsu T 2013 Defensive bacteriome symbiont with a drastically reduced genome. Curr Biol 23:1478–1484. doi:10.1016/j.cub.2013.06.027. 23850282

[26] Miller IJ, Rees ER, Ross J, Miller I, Baxa J, Lopera J, Kerby RL, Rey FE, Kwan JC 2018 Autometa: automated extraction of microbial genomes from individual shotgun metagenomes. bioRxiv doi:10.1101/251462. PMC6547426 30838416

